# DNA Methylation at a Bovine Alpha Satellite I Repeat CpG Site during Development following Fertilization and Somatic Cell Nuclear Transfer

**DOI:** 10.1371/journal.pone.0055153

**Published:** 2013-02-01

**Authors:** Christine Couldrey, David N. Wells

**Affiliations:** Animal Productivity, AgResearch Ruakura Research Centre, Hamilton, New Zealand; University of Connecticut, United States of America

## Abstract

Incomplete epigenetic reprogramming is postulated to contribute to the low developmental success following somatic cell nuclear transfer (SCNT). Here, we describe the epigenetic reprogramming of DNA methylation at an alpha satellite I CpG site (αsatI-5) during development of cattle generated either by artificial insemination (AI) or *in vitro* fertilization (IVF) and SCNT. Quantitative methylation analysis identified that SCNT donor cells were highly methylated at αsatI-5 and resulting SCNT blastocysts showed significantly more methylation than IVF blastocysts. At implantation, no difference in methylation was observed between SCNT and AI in trophoblast tissue at αsatI-5, however, SCNT embryos were significantly hyper-methylated compared to AI controls at this time point. Following implantation, DNA methylation at αsatI-5 decreased in AI but not SCNT placental tissues. In contrast to placenta, the proportion of methylation at αsatI-5 remained high in adrenal, kidney and muscle tissues during development. Differences in the average proportion of methylation were smaller in somatic tissues than placental tissues but, on average, SCNT somatic tissues were hyper-methylated at αsatI-5. Although sperm from all bulls was less methylated than somatic tissues at αsatI-5, on average this site remained hyper-methylated in sperm from cloned bulls compared with control bulls. This developmental time course confirms that epigenetic reprogramming does occur, at least to some extent, following SCNT. However, the elevated methylation levels observed in SCNT blastocysts and cellular derivatives implies that there is either insufficient time or abundance of appropriate reprogramming factors in oocytes to ensure complete reprogramming. Incomplete reprogramming at this CpG site may be a contributing factor to low SCNT success rates, but more likely represents the tip of the iceberg in terms of incompletely reprogramming. Until protocols ensure the epigenetic signature of a differentiated somatic cell is reset to a state resembling totipotency, the efficiency of SCNT is likely to remain low.

## Introduction

The process of cloning by SCNT requires the epigenetic signature of a differentiated somatic cell to be reset to a state resembling totipotency, capable of driving full development after fusion of the cell with an enucleated oocyte cytoplast. Because the genomic sequence of the donor cell theoretically remains unchanged throughout this process, incomplete nuclear reprogramming is widely postulated to be a major contributor to the low developmental success rate following SCNT. The field of nuclear reprogramming is filled with contradictory reports. Even if investigations are limited to a subset of epigenetic modifications such as DNA methylation, a broad spectrum of findings relating to the efficiency of epigenetic reprogramming following SCNT has been published. Varying combinations of hypo-methylation [1], [2], [3], [4], [5], [6], hyper-methylation [7], [8], [9], [10], mosaic methylation states [11] and normal methylation [8], [12], [13], [14], [15], [16], [17] following SCNT have been reported over the past decade. Direct comparison of these studies is problematic due to differences in analytical methods, genomic regions and tissue types investigated. However, data reported within individual studies have also often shown both correct and inappropriate reprogramming within a single animal, depending on the genomic regions examined [8], [14], [15].

Previous work from our laboratory identified correct DNA methylation at 174/175 CpG sites across 10 genomic sequences analyzed in the sperm of bulls produced by SCNT compared with sperm from control bulls generated by natural fertilization [15]. The one CpG site (termed αsatI-5 here) that exhibited significantly different methylation in the sperm from control and SCNT bulls was located within the repetitive alpha satellite I sequence. This CpG site is present in thousands of tandemly repeated copies in the bovine genome forming a major component of eukaryotic centromeric heterochromatin, the functions of which include ensuring faithful segregation of chromosomes during cell division (review [18]). Because of the vital importance of correct chromosomal segregation during cell division [19], the CpG site found to be differentially methylated in sperm from control and SCNT bulls was examined during the development of embryos, fetuses, and post-natal animals generated by fertilization and SCNT. Particular attention was paid to tissues that have regularly shown gross abnormalities during post-mortem examination of SCNT fetuses and animals, such as placenta, kidney, adrenal and muscle [20], [21]. Here we describe a time course series following the epigenetic reprogramming of DNA methylation at an alpha satellite I CpG site during cattle development as a model CpG site for following incomplete reprogramming following SCNT.

## Results

**Figure 1:**
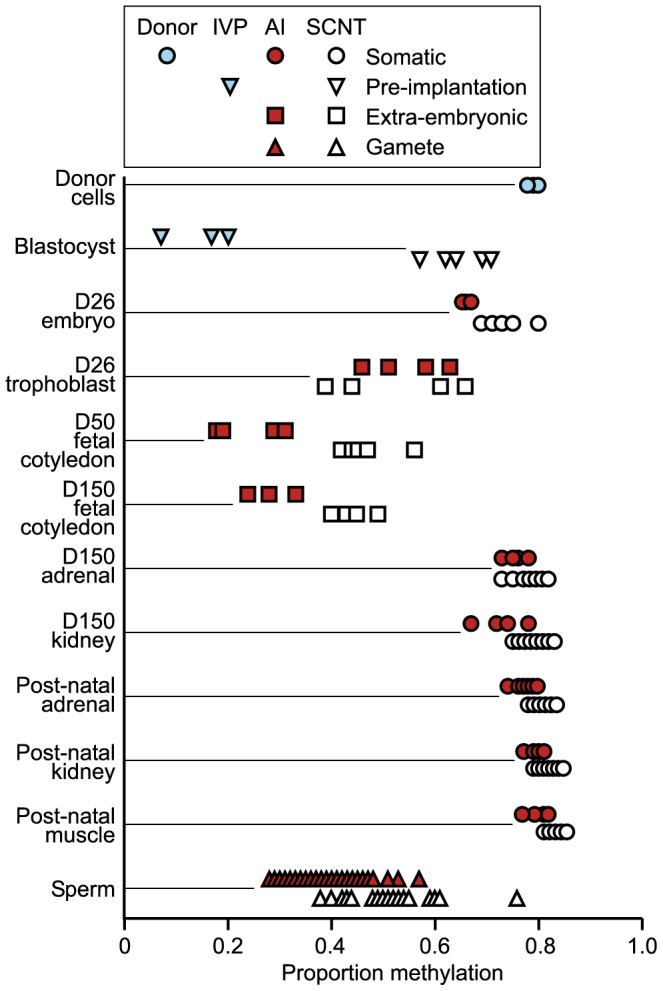
Proportion of DNA methylation. Proportion of methylation at the alpha satellite I sequence CpG site analyzed during embryonic, fetal and post-natal development from SCNT and control tissues. All control samples were generated using AI, except for the blastocyst stage embryos that were generated using IVF. Blastocysts were analyzed as pools of 10 embryos while all other samples were analyzed on an individual basis.

The αsatI-5 CpG site during development displayed high levels of DNA methylation in somatic tissues and SCNT blastocysts; with lower levels in extra-embryonic tissues, male gametes (sperm), and IVF blastocysts. SCNT tissues and blastocysts showed a significantly higher proportion of methylation than corresponding AI tissues and IVF blastocysts in all groups, except peri-implantation trophoblast (Figure 1).

SCNT donor cells were highly methylated (average proportion = 0.79) and the resulting SCNT blastocysts showed significantly more methylation (average proportion methylation = 0.65) at αsatI-5 than IVF blastocysts (average proportion = 0.14) (P = 2.81E−05) (Figure 1). This difference (0.51) in the proportion of DNA methylation between SCNT and control embryos was the largest difference observed in this time course study. Despite this very significant difference in DNA methylation, both IVF and SCNT blastocysts showed a lower proportion of DNA methylation than sperm used for fertilization and donor cells, respectively.

The differences in methylation levels observed at the blastocyst stage were significantly reduced in the 19 day period between the time of blastocyst transfer into recipient heifers and early embryo implantation into the uterus. At a peri-implantation time point on Day 26, no difference was observed in the proportion of αsatI-5 copies methylated in the trophoblast tissue. However, at this same time point, the proportion of DNA methylation in the developing embryo proper was still significantly greater in SCNT compared to AI (P = 0.004), although the magnitude of the difference had decreased from 0.51 in the blastocyst to 0.08 in the peri-implantation embryo proper.

Following implantation, DNA methylation of αsatI-5 in placental tissues from fetuses produced by AI decreased until the fetal cotyledons were fully formed. On average, some demethylation occurred at αsatI-5 in placental tissue of fetuses generated by SCNT, however, on an individual level the proportion of methylation in mid-gestation (D 150) fetal cotyledons from SCNT fetuses were all within the same range as in SCNT peri-implantation trophoblast tissue (Figure 1). The lack of SCNT extra-embryonic demethylation at αsatI-5 resulted in a mean difference in the proportion of methylation of 0.16 in mid-gestation fetal cotyledons between SCNT and AI tissues.

In contrast to placental tissues, the proportion of methylation at αsatI-5 remained high in somatic tissues during fetal and post-natal development (Figure 1). Differences in the proportion of methylation were smaller in somatic tissues than placental tissues (0.03–0.06), but on average, all SCNT tissues examined showed statistically significant hyper-methylation (P = 1.8E−05– P = 0.04).

In adult bulls, the average proportion of methylation measured at αsatI-5 in sperm was lower than in somatic tissues. The average hyper-methylation previously published in the sperm from cloned males [15] continued to be observed when an increased number of samples were analyzed in order to determine the variation of methylation in sperm from control and SCNT produced bulls (control n = 106, SCNT n = 23).

On average, all fetal and post-natal SCNT somatic tissues were hyper-methylated compared to those obtained from fetuses and cattle generated by AI; however, at an individual animal level a considerable overlap existed in the proportion of methylation at this sequence, with some SCNT individual animals exhibiting apparently normal DNA methylation levels. In contrast, the proportion of methylation in post-implantation placental tissue was clearly different in animals that had been generated by AI or SCNT, with no overlap in methylation levels detected at either 50 or 150 days of gestation in the animals measured.

Analysis of variance of the methylation by breed showed that there were no significant differences among the breeds in any of the tissues or time points examined (data not presented).

Methylation data from surrounding CpG sites able to be analyzed simultaneously by Sequenom analysis (Figure S1 and Table S1) showed that CpGs close to αsatI-5 showed some similarity to the data reported in this manuscript, while more distant CpGs showed progressively less similarity to αsatI-5.

## Discussion

The decrease in the proportion of methylation at αsatI-5 in SCNT embryos during the first seven days of development, from the donor cell state, followed by continued decrease in average methylation in extra-embryonic tissues, together with the lower levels of methylation found in sperm of SCNT bulls demonstrates that some epigenetic reprogramming is indeed occurring following SCNT. However, comparison of the methylation levels measured in SCNT blastocysts with those generated by IVF suggests that the seven day period from SCNT to blastocyst formation is either an insufficient period of time for complete reprogramming, or that SCNT embryos do not have the appropriate reprogramming factors to ensure that this repeat region is appropriately reprogrammed by the blastocyst stage. Similar findings have previously been reported in other repeat regions [10], [22]. Although the repeat region analyzed does not encode proteins, the transcription of non-coding RNAs from this sequence has been shown to play an integral role in maintaining chromatin in a specific conformation (review [18]) and aberrant RNA transcription of satellite sequences has been correlated with excessive DNA demethylation and genomic instability [23]. Inappropriate DNA methylation at Satellite II sequence has been associated with missegregation of chromosomes in human leucocytes [24] suggesting a role for DNA methylation in repetitive centromeric sequence, together with histone modifications [25], in ensuring faithful chromosome segregation. The presence of hyper-methylation in these, often centrometic, repeat regions in SCNT embryos would therefore be expected to cause chromatin to be more condensed than in embryos generated by sperm fertilization of oocytes and may lead to a widespread lack of transcription that has been observed previously [26], [27] as well as potentially disrupting correct maintenance of sister chromatic cohesion. Both of these scenarios may in turn contribute, either immediately, or later in development, to the inefficiency of producing live offspring by SCNT. Recent studies in mouse SCNT embryo development have identified that disruption of chromosomal segregation is widespread following SCNT and its occurrence prior to the 8 cell stage significantly inhibits post-implantation development [19], thus providing strong evidence that maintenance of correct chromosomal structure is vital for normal development.

The decreasing levels of DNA methylation at the αsatI-5 CpG site in placental tissues from fetuses generated by AI supports previously published studies that have reported, at least in mice, lower levels of global DNA methylation in extra-embryonic tissues compared with embryonic tissues (review [28]). The decrease of DNA methylation observed in placental tissues during development is suggestive of passive demethylation occurring with repeated cycles of DNA synthesis and cell division at αsatI-5. However, if demethylation is occurring in a passive manner in placental tissues from AI derived fetuses, it is unclear why the corresponding tissues from fetuses generated by SCNT fail to lose DNA methylation during the same period.

It is known that placental tissues of SCNT pregnancies display abnormalities. Fetal cotyledons from SCNT pregnancies are larger in size but fewer in number compared with AI generated pregnancies [20], [29] and it is possible that this phenotypic abnormality is linked with the epigenetic abnormality reported here. Unfortunately, it is currently impossible to determine whether inappropriate DNA methylation is the cause, or the effect of the phenotype.

Although the average methylation levels observed in somatic tissues from AI and SCNT mid-gestation fetuses and post-natal animals were all significantly different, the absolute differences in average methylation levels between the groups were relatively small (proportion methylation differences of only 0.03–0.06). The data presented here show that the analyzed somatic tissues have similar methylation levels at αsatI-5 as fibroblasts and ovarian follicular cells in culture. This data also suggests that more normal methylation is achieved in tissues more similar to the donor cell used for SCNT because either a) somatic cells have strong epigenetic memory or b) tissues in which less change in methylation state is required, appear to have more normal methylation acquired in a passive manner; that is, DNA methylation levels are simply maintained because no change is needed.

The small differences in the average proportion of methylation between SCNT and AI somatic tissues, in the range of 0.03–0.06, make it challenging to imagine how these might lead to biological effects. However, as αsatI-5 is present in thousands of copies in all autosomal chromosomes in cattle [30], it is possible that some alpha satellite I repeats are appropriately methylated, while other repeats are more hyper-methylated than the overall proportion of methylation measured here in individual samples suggests. Alternatively, somatic tissue cellular composition may be subtly different in SCNT compared to AI derived fetuses and post-natal animals. If this were the case, the uniformity of the differences in the proportion of methylation observed across the different tissues and time points analyzed would be hard to explain given the different cellular make up of these complex and highly specialized tissues.

Often epigenetic analysis focuses on comparisons of mean values for groups. The data presented here show that on average the SCNT animals have statistically greater levels of DNA methylation than AI animals at this CpG site. However, on an individual animal basis, this study also clearly illustrates that many cloned animals have DNA methylation levels in somatic tissues and male gametes that fall well within the normal range. This once again highlights the fact that many cattle produced by SCNT are “apparently normal” [31], [32], [33], [34]. To date, no correlation between DNA methylation and gross phenotype has been observed in those SCNT fetuses or animals that have been shown, upon post-mortem to have abnormal development such as hydroallantois, or inappropriate kidney, adrenal or muscle organization and growth ([20], [21] Couldrey, unpublished data). Unfortunately, a major limitation of these studies is that repeated measurements of DNA methylation cannot be made from a single individual in order to track methylation levels during development. The measurements made are therefore a snapshot of DNA methylation at a single time point for each animal. It is highly likely that differences in epigenetic states early in development may set up an abnormal developmental path, even if those epigenetic differences are no longer seen in tissues showing abnormalities. It must also be remembered that DNA methylation is not the only epigenetic modification able to alter DNA structure. Recent studies have also indicated the potential involvement of hydroxymethylation. Unfortunately, to date, it is not possible to measure hydroxymethyaltion at a single nucleotide resolution and we are therefore unable to distinguish if the levels measured here are due to DNA methylation or hydroxymethylation.

This developmental time course study indicates that epigenetic reprogramming does occur, at least to some extent, following SCNT. However, when epigenetic changes of a large magnitude are required, SCNT tissues are less able to accomplish this than tissues from AI generated embryos/fetuses. Although many CpG sites are reset correctly [14], [15], some appear to have an “epigenetic memory” [35]. Perhaps it is these often subtle, but persistent errors in the epigenomes of SCNT animals that accumulate in a stochastic manner and contribute to the limited success of SCNT. Until technologies have been developed that can ensure appropriate epigenetic status from the first cell division onwards, the efficiency of SCNT is likely to remain low.

## Materials and Methods

### Animal Studies

All animal manipulations were conducted in accordance with the regulations of the New Zealand Animal Welfare Act of 1999 and the Ruakura Animal Ethics Committee (AE Applications 10067 and 11263). Animal use was under the three R’s - i.e. reduction, replacement, refinement. Cows raised for commercial slaughter carrying the peri-implantation or mid-gestation fetuses analyzed were sacrificed using standard commercial captive bullet procedures at the Ruakura abattoir. Animals undergoing post-natal analysis were sacrificed by the administration of sodium pentabarbitone under veterinary supervision. All animals were visually monitored on a daily basis for changes in behaviour and cared under the rules for the code of recommendations and minimum standards for the care and use of animals for scientific purposes http://www.biosecurity.govt.nz/regs/animal-welfare/research.

### Donor Cells

Donor somatic cells were cultured in DMEM/F12 with 10% FCS, as described previously [29]. Three donor cell lines were used, a fibroblast cell line from a genetically superior Friesian bull, a fibroblast cell line from a Limousin×Jersey steer found to have desirable carcass composition upon slaughter, and an ovarian follicular cell line from a high performing Friesian cow.

### Embryo Production

SCNT and IVF embryos were generated as previously described [29]. Donor cells used were obtained from a fibroblast cell line derived from a high genetic merit Friesian bull. Day 7 blastocysts were grouped into pools of 10 for analysis: IVF n = 3 pools, SCNT n = 5 pools. Individual peri-implantation embryos (day 26) were separated into a) the embryo proper and b) the trophoblast (with yolksac and allantois removed) before being analyzed singularly (embryo proper: AI n = 5, SCNT n = 5; trophoblast: AI n = 4, SCNT n = 4).

### Production and Collection of Fetal and Post-natal Tissues

After in vitro culture for seven days, the SCNT embryos were transferred to synchronized recipients. Control pregnancies were generated by AI with frozen semen from the bull which provided the donor cells or genetically similar bulls. For sample collection, the uteri and its contents were recovered after slaughter of the recipient dams. Extra-embryonic tissues were collected from a single representative fetal cotyledon from SCNT and AI samples at a) the earliest timepoint feasible for collecting cotyledon burrs which is day 50 of pregnancy (day 50 fetal cotyledon: AI n = 5, SCNT n = 5) and at a timepoint at which many SCNT pregnancies fail due to hydroallantois (day 150) (mid-gestation fetal cotyledon: AI n = 4, SCNT n = 5). Gross fetal and placental morphology was recorded and fetal and placental tissue samples collected and snap-frozen in liquid nitrogen. Two organs often showing gross abnormalities, namely the kidney and adrenal glands, were collected at mid-gestation (kidney: AI n = 4, SCNT n = 14; adrenal: AI n = 4, SCNT n = 14). During post-natal necropsy at three to six months of age, three organs often showing gross abnormalities were collected, namely the kidney, adrenal glands and muscle (kidney: AI n = 7, SCNT n = 21; adrenal: AI n = 7, SCNT n = 14; muscle: AI n = 4, SCNT n = 7).

### Semen Collection

Semen was collected from bulls using standard commercial practice. Control sperm samples were obtained from a total of 106 conventionally bred bulls, including the three Friesian bulls that had been used as SCNT donors prior to or during this study (one of which provided a donor cell line) and the sire of the female donor cell line. The remaining control bulls were of varying breeds: Friesian (n = 15), Jersey (n = 5), Friesian×Jersey (n = 15), Blond d’Aquitaine (10), Limousin (n = 8), Hereford (n = 11), Shorthorn (n = 8), Angus (n = 17), Maine Anjou (n = 5), and Pie Rouge (n = 8). SCNT semen samples were obtained from 23 cloned bulls generated from five independent Friesian or Limousin×Jersey donor cell lines. In all cases, all cells collected in the ejaculate were used.

### DNA Extraction

Semen: DNA was extracted from approximately 250 µl of semen as described previously [15]. Donor cells: DNA was extracted from approximately 10^6^ cells using standard proteinase K digestion followed by ethanol precipitation. Somatic and extra-embryonic: tissues were ground to a powder in liquid nitrogen to ensure homogeneity for DNA sampling. Between 20 and 100 mg of tissue was then used for DNA extraction using proteinase K digestion followed by ethanol precipitation. Pools of blastocysts were lyzed and treated briefly with proteinase K using the EZ-DNA methylation direct kit (Zymo, CA, USA) following manufacturers’ instructions.

### Primer Design

Primers were designed using MethPrimer to amplify the CpG island in repetitive DNA sequence alpha satellite I (Genbank AJ293510) after bisulfite treatment of DNA as described [14].The CpG site of interest identified in previously published work [15] is illustrated in bold and underlined in the alpha satellite I region amplified: CCCCTCCTGATCTGGACAGGAGGGTCGACTCCCCTGCTTTGTCTGGAAGGGGTTCCCGACCTTCCGGTCGCACCTCAGGATGAGGC**CG**GTCTCACGAAGACATTCCAGACGTGGCCTCGTGGGTGGTTCCACATTCCGTAGGACCCCGATTTCCCGGTCCCCTCTTGATAAGAACCCGATGCCCGGACACCTCTCGAACTCCAGCCTGTGAATGAAGTCAACACGAAGGGGCAGTTTTTCCGTGCATCGATCGGAAAAACCCCTGGTTTCAAATACAGC.

### DNA Methylation Analysis

DNA samples were analyzed using Sequenom MassARRAY technology as previously described [14], [15], [36]. Briefly, the EZ-DNA methylation direct kit was used to produce methylation-dependent sequence variations of C to T and regions of interest were amplified using T7 tagged PCR primers in triplicate. Replicates were pooled for analysis to minimize bias and variation as described by Coolen et. al. 2007 [36]. *In vitro* amplification and transcription was performed on the reverse strand with simultaneous U specific cleavage by RNAseA. Samples were subject to mass spectrometry to provide high-resolution DNA methylation analysis, quantitative to 5% methylation for informative CpG dinucleotides [36]. For each PCR and subsequent MassARRAY analysis no DNA controls were included together with positive controls of known methylation levels to ensure consistency of results from assay to assay.

### Statistical Analysis

Spectra were analyzed using proprietary peak picking and signal-to-noise ratio calculations. The relative methylation of the CpG site was then calculated (EpiTYPER, Sequenom, CA, USA) by dividing the peak intensity (area under the peak) of the fragment representing the original methylated DNA, by the sum of the intensities of the peaks representing both methylated and non-methylated DNA. Significance between AI and SCNT samples for each tissue and average methylation difference in control verses SCNT samples were analyzed by *t*-test. Analysis of variance was used to determine whether DNA methylation at this CpG site was significantly different between breeds.

## Supporting Information

Figure S1 **Average DNA methylation at CpG sites surrounding and including αsatI-5 DNA sequence.** Although not all CpG sites in the αsatI sequence are able to be measured (or measured individually), DNA methylation levels for those sites that could be measured are presented as averages in each tissue analyzed as a comparison with αsatI-5.
(TIF)

Table S1 **Statistical significance of differences in DNA methylation.** T-test P values for comparison of DNA methylation levels between control (IVF and AI) compared with SCNT embryos, extra-embryonic, fetal and post-natal tissues in the αsatI sequence including αsatI-5. In this instance a P value of 1.000 indicates that all samples in both groups showed 100% methylation at this site. Red text indicates P value is <0.001, blue text indicates P = 0.001- 0.05, black text indicates insignificant.
(DOCX)
