# Supplementary figures and images for: DNA Methylation at a Bovine Alpha Satellite I Repeat CpG Site during Development following Fertilization and Somatic Cell Nuclear Transfer
Source: PLoS One. 2013 Feb 1;8(2):e55153. doi: 10.1371/journal.pone.0055153 (PMC3562336; doi:10.1371/journal.pone.0055153)

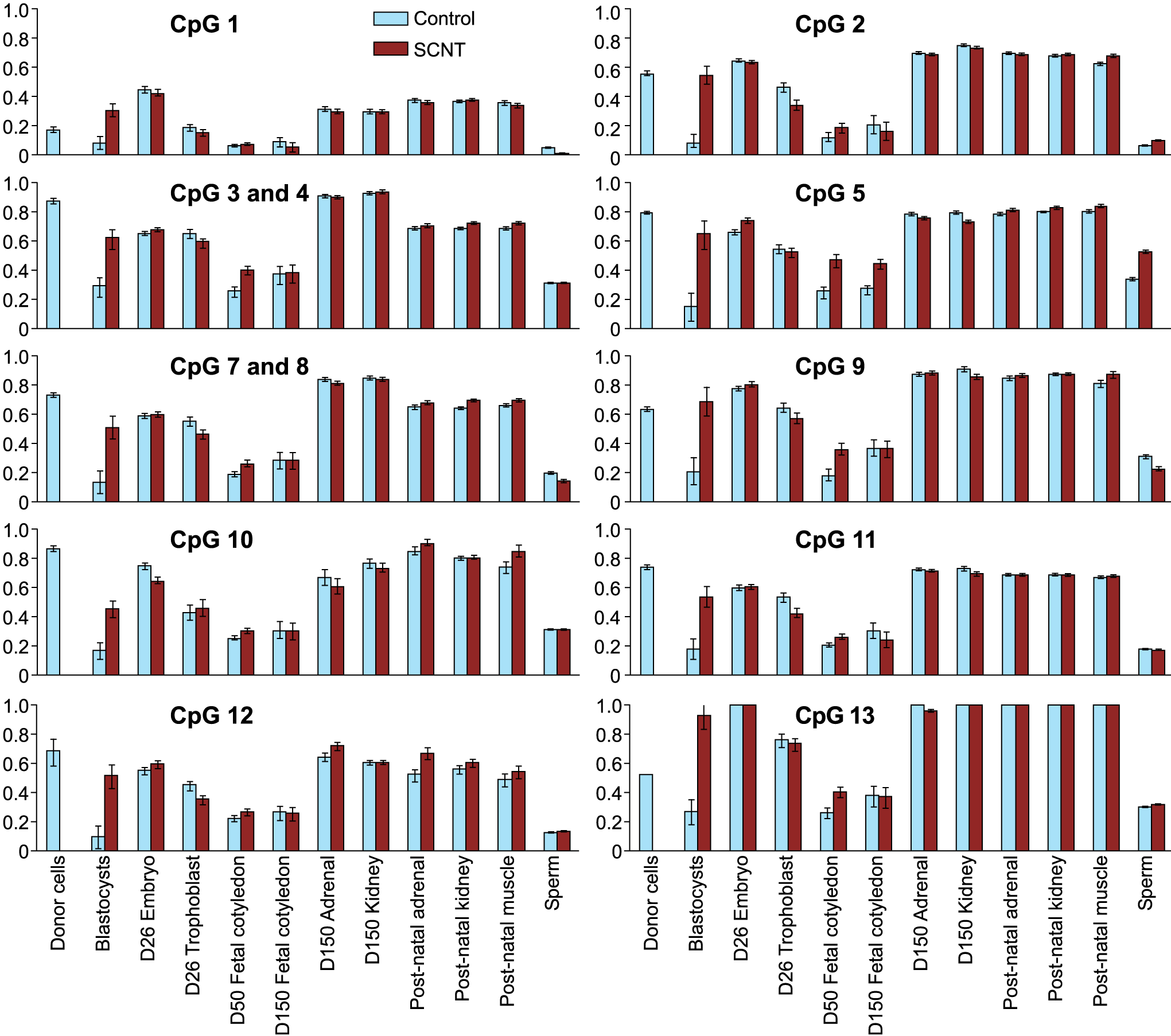

Supplement: Figure S1 — Average DNA methylation at CpG sites surrounding and including αsatI-5 DNA sequence. Although not all CpG sites in the αsatI sequence are able to be measured (or measured individually), DNA methylation levels for those sites that could be measured are presented as averages in each tissue analyzed as a comparison with αsatI-5. (TIF) [file pone.0055153.s001.tif]
