# Supplementary material for: DNA Methylation at a Bovine Alpha Satellite I Repeat CpG Site during Development following Fertilization and Somatic Cell Nuclear Transfer
Source: PLoS One. 2013 Feb 1;8(2):e55153. doi: 10.1371/journal.pone.0055153 (PMC3562336; doi:10.1371/journal.pone.0055153)
Supplement: Table S1 — Statistical significance of differences in DNA methylation. T-test P values for comparison of DNA methylation levels between control (IVF and AI) compared with SCNT embryos, extra-embryonic, fetal and post-natal tissues in the αsatI sequence including αsatI-5. In this instance a P value of 1.000 indicates that all samples in both groups showed 100% methylation at this site. Red text indicates P value is <0.001, blue text indicates P = 0.001- 0.05, black text indicates insignificant. (DOCX) [file pone.0055153.s002.docx]

|  | 1 | 2 | 3.4 | 5 | 7.8 | 9 | 10 | 11 | 12 | 13 |
| --- | --- | --- | --- | --- | --- | --- | --- | --- | --- | --- |
| Blastocysts | 0.000 | 0.000 | 0.000 | 0.000 | 0.001 | 0.000 | 0.002 | 0.000 | 0.000 | 0.000 |
| D26 Embryo | 0.626 | 0.545 | 0.092 | 0.004 | 0.570 | 0.278 | 0.024 | 0.847 | 0.509 | 1.000 |
| D26 Trophoblast | 0.061 | 0.015 | 0.037 | 0.086 | 0.036 | 0.036 | 0.775 | 0.016 | 0.047 | 0.128 |
| D50 Fetal cotyledon | 0.738 | 0.047 | 0.007 | 0.000 | 0.040 | 0.005 | 0.080 | 0.070 | 0.266 | 0.034 |
| D150 Fetal cotyledon | 0.522 | 0.599 | 0.943 | 0.000 | 0.985 | 0.971 | 0.938 | 0.552 | 0.925 | 0.952 |
| D150 Adrenal | 0.607 | 0.742 | 0.360 | 0.040 | 0.173 | 0.811 | 0.611 | 0.704 | 0.327 | 0.059 |
| D150 Kidney | 0.957 | 0.467 | 0.330 | 0.001 | 0.641 | 0.122 | 0.685 | 0.200 | 0.937 | 1.000 |
| Post-natal adrenal | 0.739 | 0.850 | 0.005 | 0.001 | 0.058 | 0.517 | 0.157 | 0.896 | 0.023 | 1.000 |
| Post-natal Kidney | 0.348 | 0.314 | 0.001 | 0.000 | 0.004 | 0.447 | 0.962 | 0.863 | 0.670 | 1.000 |
| Post-natal muscle | 0.453 | 0.057 | 0.005 | 0.006 | 0.036 | 0.104 | 0.146 | 0.730 | 0.560 | 1.000 |
| Sperm | 0.114 | 0.014 | 0.887 | 0.000 | 0.168 | 0.426 | 0.751 | 0.576 | 0.739 | 0.496 |
